# Generation of single photons with highly tunable wave shape from a cold atomic ensemble

**DOI:** 10.1038/ncomms13556

**Published:** 2016-11-25

**Authors:** Pau Farrera, Georg Heinze, Boris Albrecht, Melvyn Ho, Matías Chávez, Colin Teo, Nicolas Sangouard, Hugues de Riedmatten

**Affiliations:** 1 ICFO-Institut de Ciencies Fotoniques, The Barcelona Institute of Science and Technology, 08860 Castelldefels, Barcelona, Spain; 2 Department of Physics, University of Basel, Klingelbergstrasse 82, 4056 Basel, Switzerland; 3 Institute for Quantum Optics and Quantum Information of the Austrian Academy of Sciences, A-6020 Innsbruck, Austria; 4 Institute for Theoretical Physics, University of Innsbruck, A-6020 Innsbruck, Austria; 5 ICREA-Institució Catalana de Recerca i Estudis Avançats, 08015 Barcelona, Spain

## Abstract

The generation of ultra-narrowband, pure and storable single photons with widely tunable wave shape is an enabling step toward hybrid quantum networks requiring interconnection of remote disparate quantum systems. It allows interaction of quantum light with several material systems, including photonic quantum memories, single trapped ions and opto-mechanical systems. Previous approaches have offered a limited tuning range of the photon duration of at most one order of magnitude. Here we report on a heralded single photon source with controllable emission time based on a cold atomic ensemble, which can generate photons with temporal durations varying over three orders of magnitude up to 10 μs without a significant change of the readout efficiency. We prove the nonclassicality of the emitted photons, show that they are emitted in a pure state, and demonstrate that ultra-long photons with nonstandard wave shape can be generated, which are ideally suited for several quantum information tasks.

## Abstract (web summary)

Generation of narrowband pure and storable single photons is an enabling step towards hybrid quantum networks interconnecting different systems. Here the authors report on a heralded single photon source based on a cold ensemble of atoms with controllable emission time and high photon shape tunability.

## Graphical abstract

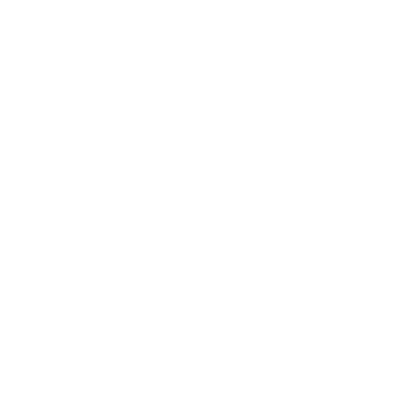

A vast range of experiments in quantum information science and technology rely on single photons as carriers of information^1^. Single photon sources are thus key components and have been continuously improved over the past years^2^. The spectrum and temporal shape of the emitted photons are important parameters of such sources^3^. The generation of ultra-long single photons is for example an essential requirement for precise interactions with media exhibiting a sharp energy structure like trapped atoms, ions or doped solids, which have been proposed as quantum memories for light^4^,^5^,^6^ and also with cavity opto-mechanical systems^7^,^8^,^9^,^10^,^11^.

Several approaches to achieve narrow linewidth photons have been investigated, including for example, cavity-enhanced spontaneous parametric down conversion^12^,^13^,^14^, cold atomic ensembles^15^,^16^,^17^,^18^,^19^,^20^,^21^,^22^, single atoms^23^,^24^, quantum dots^25^ or trapped ions^26^,^27^. Moreover, significant efforts have been devoted to generate single photons with tunable temporal shapes^20^,^26^,^28^,^29^,^30^,^31^,^32^,^33^,^34^, which is important for many applications in quantum information science^35^,^36^. However, most of the previous approaches offered only a limited tuning range of the photon duration up to at most one order of magnitude^20^,^26^,^32^.

In this paper, we demonstrate a single photon source with a wide tuning range of three orders of magnitude, up to single photon durations of 10 μs. To our knowledge, this represents the longest photons generated from an atomic ensemble. Our source is based on a cold atomic ensemble quantum memory (QM) following the scheme of Duan, Lukin, Cirac, and Zoller (DLCZ)^37^, which allows us to release the single photons on demand after a programmable delay. This is essential for temporal synchronization tasks as for example needed for quantum repeater architectures^38^,^39^ or synchronization of photon pair sources^40^. In contrast to most former DLCZ experiments, we apply readout pulses with very flexible temporal shapes, which are accurately controlled over several orders of magnitude in amplitude and time. This enables the generation of ultra-long single photons with very flexible wave shapes and coherence times much longer than the lifetime of the involved excited state. We characterize the emitted photons by measuring their heralded and unheralded autocorrelation functions, demonstrating a high degree of anti-bunching and purity.

## Results

### Heralded single photon source with controllable emission time

**Figure 1:**
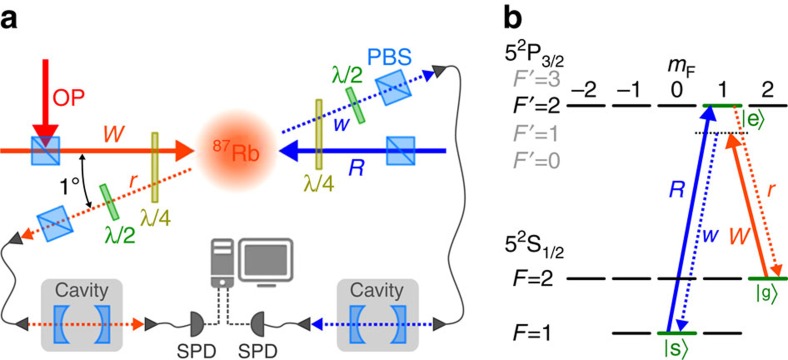
Experimental set-up and level scheme. (**a**) Experimental set-up. Write pulse (*W*) and read pulse (*R*) are sent counterpropagating into the atomic cloud. Write and read photons (denoted by *w* and *r*) are sent after polarization filtering via fibres to frequency filtering cavities before being detected by SPDs. (**b**) Energy levels of the D2 line of ^87^Rb and coupling scheme for the DLCZ experiment.

Our heralded single photon source is based on a cold ensemble of *N* identical ^87^Rb atoms in a magneto-optical trap. Each atom exhibits a Λ-type level scheme consisting of a ground state |*g*〉=|5^2^*S*_1/2_, *F*=2, *m*_*F*_=2〉 a storage state |*s*〉=|5^2^*S*_1/2_, *F*=1, *m*_*F*_=0〉 and an excited state |*e*〉=|5^2^*P*_3/2_, *F*=2, *m*_*F*_=1〉 (see Fig. 1b). The atoms are initially prepared in the ground state |*g*〉 by optical pumping. A weak write pulse, detuned from the |*g*〉→|*e*〉 transition, probabilistically creates a delocalized single-collective spin excitation (spin-wave) in the memory by transferring a single atom into the |*s*〉 state. This process is heralded by a Raman scattered write photon. The state of the spin-wave is to first order given by


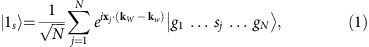


where **x**_*j*_ denotes the spatial position of the *j*th atom and **k**_*W*_ and **k**_*w*_ are the wave vectors of the write pulse and the write photon, respectively. Neglecting noise, the joint state of the write photon and the associated spin-wave is described by a two-mode squeezed state as


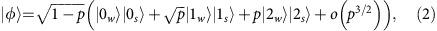


with *p* the probability to create a spin-wave correlated with a write photon in the detection mode. After a programmable delay, the spin-wave is converted back to a single read photon by a read pulse, which is resonant with the |*s*〉→|*e*〉 transition. Due to collective interference of all atoms, the read photon is emitted in a well defined spatial mode given by the phase matching condition **k**_*r*_=**k**_*R*_+**k**_*W*_−**k**_*w*_, where **k**_*R*_ and **k**_*r*_ are the wave vectors of the read pulse and read photon, respectively. The noise-corrected retrieval efficiency is defined as *η*_ret_=(*p*_*w*,*r*_−*p*_*w*,*nr*_)/*p*_*w*_, where *p*_*w*,*r*_ is the probability to detect a coincidence between a write and a read photon, *p*_*w*,*nr*_ is the probability to detect a coincidence due to background noise and *p*_*w*_ is the probability to detect a write photon per trial.

### Experimental set-up

**Figure 2:**
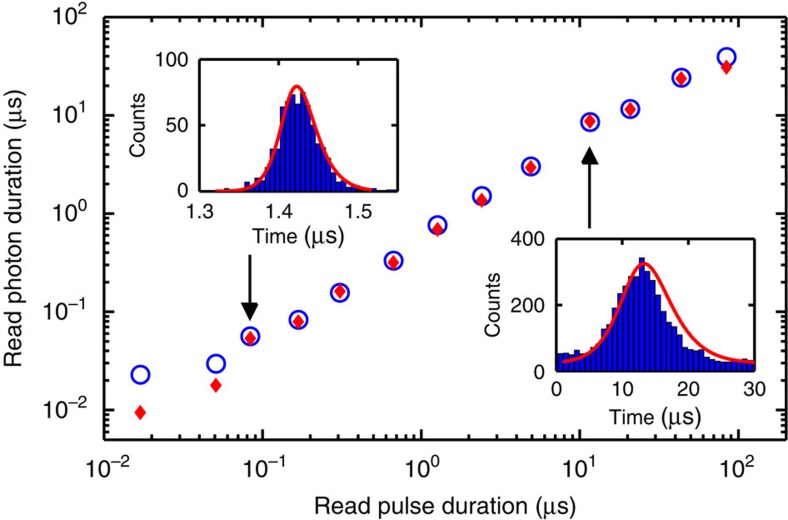
Temporal duration of the read photon versus the duration of the driving read pulse. Experimentally measured durations (FWHM) (blue circles, errorbars smaller than symbol size) are compared with numerical simulations (red diamonds). The insets show two examples of the read photon wave shape as reconstructed from the number of counts and arrival times in the SPDs (blue histograms) as well as the simulated wave shapes (red lines) for which we allowed at most 10% adjustment of the input parameters to account for experimental inaccuracies.

The experimental set-up is shown in Fig. 1a. All light beams are derived from diode lasers resonant to the *D*_2_ line of ^87^Rb at 780 nm. To generate the desired laser pulses, the beams are modulated by acousto-optic modulators in double-pass configuration driven by an arbitrary waveform generator (Signadyne AWG-H3384) with a sampling frequency of 1 GS s^−1^ and amplifiers (AA Optoelectronic AMPA-B-34). We combine a magnetic gradient of 20 G cm^−1^ with cooling light (red detuned from the |*F*=2〉→|*F*′=3〉 transition) and repumping light (resonant with the |*F*=1〉→|*F*′=2〉 transition) to load *N*≈10^8^ Rubidium atoms into the MOT. After a 1.6 ms long optical molasses phase, we prepare all population in the |*g*〉 Zeeman sublevel by applying repumping light and *σ*^+^ polarized optical pumping (OP) light on the |*F*=2〉→|*F*′=2〉 transition. The spin-wave is generated by sending a write pulse of 15 ns duration (full-width at half-maximum, FWHM), which is red detuned by 40 MHz from the |*g*〉→|*e*〉 transition. The heralding write photon is collected at an angle of 1° with respect to the write/read pulse axis. By changing the intensity of the write pulse, we can adjust the probability *p*_*w*_ to detect a write photon per trial. For the experiments presented in this paper, *p*_*w*_ ranges between 0.25% and 1% depending on the measurement. The read pulse, counterpropagating with the write pulse, is resonant with the |*s*〉→|*e*〉 transition and its temporal shape can be precisely controlled. The read photon is collected in the same spatial mode but opposite direction of the write photon. By measuring the transmission of classical light sent through the photons axis and by comparison of experimental and theoretical data in Figs 2 and 5, we infer a coupling efficiency of the read photon into the first fibre of *η*_fibre_≈60%. The polarization of the write and read pulses in the frame of the atoms is *σ*^−^ and *σ*^+^, respectively, while the detected write and read photons are *σ*^+^ and *σ*^−^ polarized. We use a combination of quarter- and half-waveplates with polarization beamsplitters to transmit only the photons with the correct polarizations. The write and read photons are moreover spectrally filtered by identical monolithic Fabry–Perot cavities with *η*_filter_≈20% total transmission (including cavity transmission and subsequent fibre coupling), before being detected by single photon detectors (SPDs) with *η*_det_=43% efficiency and a dark count rate of 130 Hz.

### Measurements

We now present the experimental results and compare them to detailed theoretical calculations. To generate read photons of variable length, we change the duration of the Gaussian-shaped read pulse as well as the storage time over several orders of magnitude (see Fig. 2). The shortest read pulse duration of ∼17 ns leads to a read photon of around 23 ns duration. After that initial data point, we observe a quite linear increase of the read photon duration with the read pulse duration up to several tens of microseconds. The lower limit of photon duration is given by the limited optical depth OD=5.5 in our experiment, which leads to limited superradiant emission of the read photon^41^. A further technical limitation is given by the finite bandwidth of the spectral filtering cavity of about 60 MHz, which additionally increases the detected duration for short read photons. This effect, together with the deviation from the adiabatic condition, partly explains the slight difference of the first data points in Fig. 2 from the theoretical prediction (see below). The upper limit of photon duration is given by the spin-wave linewidth, which is mainly determined by thermal atomic motion and spurious external magnetic fields. This currently limits the maximal storage time in the memory to about 50 μs (see Supplementary Note 1 and Supplementary Fig. 1). In addition, the photon duration will also be limited by the coherence time of the read laser, which has a specified linewidth of 20 kHz. However, within the above limits, we demonstrate that the photon duration is fully tunable and that the Gaussian wave shape of the driving read pulse is preserved in the readout process (see insets).

The dynamics of the write and read photon pairs is modelled using the Heisenberg–Langevin equations. For slowly varying optical fields propagating in a pencil-shape atomic ensemble, explicit expressions for both the write and read photon fields can be obtained in the adiabatic approximation^42^. These field expressions can be subsequently used to reproduce the read photon emissions conditioned on the detection of a write photon from first and second order correlation functions (see Supplementary Note 2). The result of these simulations which are based on independent measurements reproduce very well the experimental data presented in Fig. 2.

**Figure 3:**
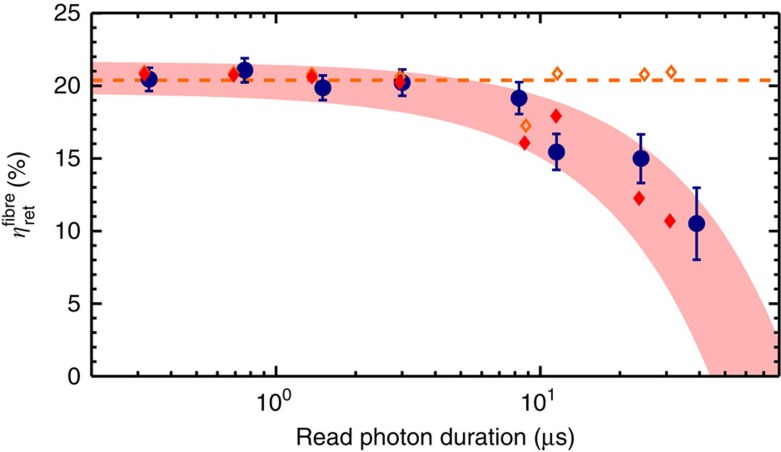
Retrieval efficiency. Fibre-coupled retrieval efficiency 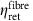 versus read photon duration (FWHM) for *p*_*w*_=0.5%. Experimental data (blue dots) are compared with numerical simulations (see Supplementary Note 2) for realistic (red diamonds) and ideal (orange diamonds) conditions. The red shaded area depicts the expected range if the input parameters of the simulation are varied by ±10%. The errorbars (±1 s.d.) correspond to the propagated Poissonian error of the photon counting statistics.

To characterize the retrieval efficiency of the photon source, we optimized the intensity of the driving read pulse for each duration (see Supplementary Note 1 and Supplementary Fig. 2). Figure 3 shows the highest achievable fibre-coupled retrieval efficiency 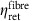
=*η*_ret_/(*η*_filter_·*η*_det_) versus the read photon duration, which corresponds to the probability of finding a read photon in the optical fibre after the vacuum cell, that is, corrected for filtering and detector efficiencies only. We observe a constant retrieval efficiency of about 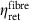
=20% up to a read photon duration of ∼10 μs. Our numerical simulations match very well with the experimental data and also show that the efficiency in the constant region is just limited by the finite OD of our atomic cloud. We verify numerically that in the absence of technical noise and considering infinite spin-wave coherence, for OD=50 an intrinsic retrieval efficiency of 80% can be achieved while maintaining control of the photon shape. The decrease of the efficiency at around 10 μs is due to dephasing of the spin-wave induced by atomic motion, spurious external magnetic field gradients^43^ and the finite read laser coherence time. In particular, our numerical simulations show clearly that in the absence of technical noise and in the limit of infinite spin coherence, the efficiency is kept constant (see orange diamonds and dashed line in Fig. 3).

**Figure 4:**
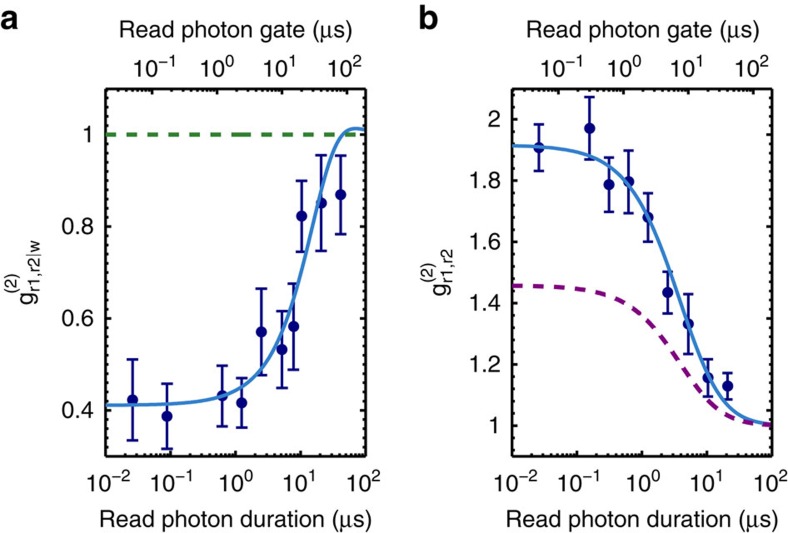
Measurement of correlation functions. Second order autocorrelation function of the generated read photons, (**a**) conditioned on the detection of a write photon in the same experimental trial at *p*_*w*_=0.25% and (**b**) not conditioned on a write photon detection at *p*_*w*_=1%. The experimental data (blue dots) are compared with a theoretical model accounting for detector imperfections, that is, a measured dark count rate of 130 Hz (blue lines). The dashed green line in (**a**) represents the classical bound of a coherent state and the dashed purple line in (**b**) shows the expected trace for a photon state with two modes. The errorbars (±1 s.d.) correspond to the propagated Poissonian error of the photon counting statistics.

Next, we characterized the state of the emitted read photons by measuring their heralded and unheralded second order autocorrelation functions depending on the read photon duration. To perform these measurements, we inserted a balanced fibre beamsplitter into the read photon arm after the spectral filtering cavity, with both output ports connected to SPDs *r*1 and *r*2. First, we recorded the autocorrelation function conditioned on the detection of a write photon, defined as^44^:


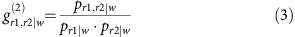


where *p*_*r*1,*r*2|*w*_ denotes the probability to measure a coincidence between both read photon detections conditioned on a write photon detection, and *p*_*r*1|*w*_, *p*_*r*2|*w*_ are the probabilities to detect a read photon via *r*1 or *r*2 conditioned on a write photon detection. The data shown in Fig. 4a clearly demonstrate the nonclassicality of the photons (that is, 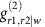
<1) up to photon durations of >10 μs. However, we do not reach the ideal value of 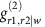
=0 of perfect single photons. For short read photon durations, we are still limited by noise due to higher-order components of the spin-wave, which can be addressed by reducing *p*_*w*_. In fact, the observed 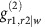
≈0.4 is consistent with former measurements at similar values for *p*_*w*_ and read pulse durations^43^. For longer read photon durations, we observe an increase of 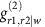, which can be simply explained by a higher number of dark counts of the SPDs for longer read photon detection gates (see upper axis in Fig. 4). The solid blue line shows the prediction of a non-perturbative theoretical model accounting for detector imperfections^45^. The agreement between the model and the experimental data is excellent.

The single mode nature of the photon state is characterized by the unconditional autocorrelation function 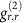 (see Fig. 4b). For an ideal two-mode squeezed state, where the write and read photons are each emitted in a single temporal mode, one expects 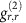
=2, which is quite well fulfilled by the measured data up to a read photon duration of roughly 1 μs. For longer durations, we observe a drop, which can be attributed to either an increasing temporal multimodality of the read photon (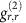 scales as 1+1/*K* with *K* denoting the number of photon modes^46^) or to measurement imperfections because of higher dark counts for larger detection gates. The solid blue line shows the theoretical prediction, assuming read photons emitted in a single mode. The excellent agreement between experiment and theory suggests that the read photons are emitted mostly in a single mode. For comparison, we also plotted the expected behaviour for a single photon with *K*=2 modes (see purple dashed line), which significantly differs from the measured data, therefore, confirming the single mode nature of the emitted read photons. Consequently, the read photons are close to being Fourier transform limited, giving linewidths ranging from around 20 MHz to <100 kHz. This, together with the conditional 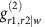, allows us to conclude that the heralded read emission is close to a pure single photon.

**Figure 5:**
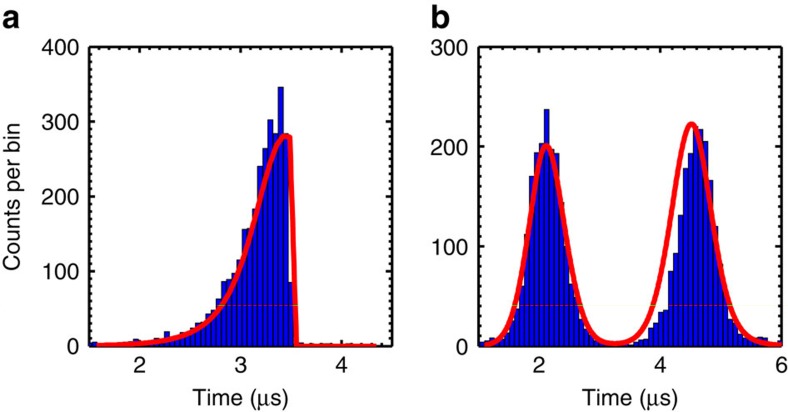
Single photons with nonstandard wave shapes. Temporal wave shape of the read photon for (**a**) a rising exponential and (**b**) a doubly peaked (time-bin) read pulse wave shape. Experimental data (blue histograms) are compared with numerical simulations (red line) for which we allowed at most 10% adjustment of the input parameters with respect to the measured data. Both histograms were taken at *p*_*w*_=0.5%.

Finally, we investigate the flexibility of the temporal shape of the generated read photons. Instead of a Gaussian-shaped read pulse, we apply read pulses with a rising exponential envelope or a doubly peaked wave shape. These two examples are important for a broad class of applications in quantum information science. Photons with rising exponential wave shape exhibit the highest possible absorbance when interacting with two-level systems^35^,^47^ and can be very efficiently loaded in optical cavities^36^,^48^. The temporal shape of the generated rising exponential read photon is shown in Fig. 5a. The driving read pulse had a 1/*e* width of 300 ns. We observe a similar retrieval efficiency of 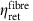
=19.8% as for a standard Gaussian-shaped pulse of same duration (*c.f.* Fig. 3). The conditioned autocorrelation function of the rising exponential photon is 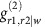
=0.31±0.14 (taken at *p*_*w*_=0.25%) and 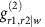
=0.73±0.12 (taken at *p*_*w*_=0.5%), which is clearly in the nonclassical regime.

As a final example, we send a doubly peaked read pulse into the prepared QM. The intensity and duration of the first readout peak was chosen such that the stored spin-wave is read out with half of the maximal efficiency and for the second peak the retrieval efficiency is maximized. This leads to a read photon with a shape shown in Fig. 5b. Photons with such a delocalized shape can be used to create time-bin qubits, which have applications in robust long-distance quantum communication^49^,^50^. The efficiency of the generated time-bin photon is 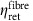
=25%, comparable to the standard Gaussian-shaped photons, and the conditioned autocorrelation function is 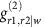
=0.54±0.11 (taken at *p*_*w*_=0.25%) and 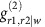
=0.75±0.08 (taken at *p*_*w*_=0.5%), which is clearly in the nonclassical regime.

## Discussion

We demonstrated a highly flexible heralded single photon source with intrinsic storage capability following the DLCZ protocol^37^ in a cold ^87^Rb ensemble. Compared with other approaches for narrowband single photon generation, such as cavity-enhanced spontaneous down conversion^12^,^13^,^14^, single atoms and ions in cavities^23^,^24^,^26^,^27^ and four-wave mixing in atomic ensembles^20^,^22^, our single photon source offers an unprecedented photon duration tunability of three orders of magnitude and the possibility to generate photons of highly flexible wave shape and an efficient emission on a single spatial and temporal mode without the need of a high-finesse cavity (see Supplementary Note 4 for a more detailed discussion).

Another important feature of our approach is that our single photon source has intrinsic storage capability, which naturally enables synchronization with other identical sources. In the following, we discuss that possibility with our current set-up. A deterministic synchronization of two such sources depends on the average time separation between successful heralding events (write photon detections) and the maximal storage time of the source. The time between heralding events depends on several parameters: First, the power of the write pulse determines the probability *p*_*w*_ and hence the detection rate of the Raman scattered write photons. However, one cannot just arbitrarily increase the write power to increase that rate because it would also lead to a degradation of the nonclassical correlations between both photons. Second, the various losses from the vacuum cell toward the final detection (mainly fibre coupling, filtering and detector efficiencies) decrease the probability and hence the rate to detect an emitted write photon quite significantly (factor ∼20). However, these are mainly technical issues, which could be improved by better equipment.

In our experiment, we typically operate the single photon source with a *p*_*w*_ of around 0.5%. Using a heralded sequence (that is, sending the read pulse only when a write photon was detected), we can generate 500 trials per 1 ms interrogation time for read photon lengths of a few microseconds, which gives an average time separation between heralding events of 400 μs. This is of course much longer than the current storage time of about 50 μs and would not allow for a deterministic synchronization of two single photon sources with the current status of the experiment.

However, note that, first, it is not necessary to be in the regime where the storage time is longer than the delay between two write photon detections to start improving the synchronization time while using the QM. The important parameter is the number of write attempts that can be done during the storage time^51^,^52^. Even with the current set-up (*N*≈25 trials per 50 μs storage time), we would reduce the synchronization time between two sources by a factor 2*N*+1≈50 compared with single shot attempts^51^. Second, with quite moderate improvements (a storage time of 1 ms^53^,^54^ and a filtering efficiency of *η*_filter_=80%), the time separation between heralding events would be 100 μs, which would be 10 times shorter than the storage time, immediately enabling the deterministic synchronization of several single photon sources.

In conclusion, we demonstrated a highly flexible single photon source with intrinsic storage capability following the DLCZ protocol^37^ in a cold ^87^Rb ensemble. By varying the temporal width of the driving read pulse, the duration of the read photons could be changed over three orders of magnitude up to several tens of microseconds. Up to a read photon duration of 10 μs, we obtain a fibre-coupled retrieval efficiency of 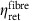
=20%, which is just limited by the OD in our experiment. We verified numerically that for OD=50 under ideal conditions, an intrinsic retrieval efficiency of 80% can be achieved while maintaining control of the photon shape. The drop in retrieval efficiency at around 10 μs is mainly due to spin-wave dephasing induced by thermal motion, which could be improved by a more sophisticated trapping of the atoms^32^,^53^. The generated read photons show a nonclassical behaviour up to durations of >10 μs for the heralded autocorrelation function and up to 1 μs we detect single photons in a pure state, currently just limited by the dark counts of our detectors. Finally, we create single photons with a nonstandard envelope like rising exponential or time-bin wave shapes, which have important applications in quantum information science. Our approach allows the generation of ultra-narrow single photons with unprecedented duration tunability and highly flexible wave shape. This will enable the interconnection of our cold atom QM with other physical systems exhibiting sharp resonances, like for example, Rb atoms prepared in a highly excited Rydberg state under the condition of EIT. Moreover, combining our approach with quantum frequency conversion techniques^53^,^55^ paves the way to the optical interconnection of the cold atom QM with different types of quantum systems, which typically demand very different photon shapes, like long-lived solid state quantum memories or opto-mechanical systems. Finally, also applying the ability to generate single photons with doubly peaked wave shapes (as shown in Fig. 5b), one could demonstrate quantum state transfer via time-bin qubits between different systems, which would be an important step toward the creation of heterogeneous quantum networks^56^.

### Data availability

The data that support the findings of this study are available from the corresponding author on request.

## Additional information

**How to cite this article:** Farrera, P. *et al*. Generation of single photons with highly tunable wave shape from a cold atomic ensemble. *Nat. Commun.* **7,** 13556 doi: 10.1038/ncomms13556 (2016).

**Publisher's note**: Springer Nature remains neutral with regard to jurisdictional claims in published maps and institutional affiliations.

## Supplementary Material

Supplementary Information Supplementary Figures 1-2, Supplementary Notes 1-4 and Supplementary References.
