# Supplementary material for: Generation of single photons with highly tunable wave shape from a cold atomic ensemble
Source: Nat Commun. 2016 Nov 25;7:13556. doi: 10.1038/ncomms13556 (PMC5133620; doi:10.1038/ncomms13556)
Supplement: Supplementary Information — Supplementary Figures 1-2, Supplementary Notes 1-4 and Supplementary References. [file ncomms13556-s1.pdf]

Supplementary Figures

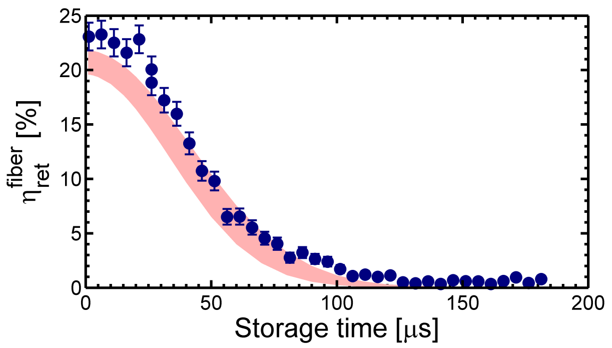

Supplementary Figure 1: **Fiber-coupled conditional retrieval efficiency $\eta_{\rm ret}^{\rm fiber}$ vs the delay between the write and read pulses.** Experimental data (blue dots) are compared with numerical simulations (red shaded area). In order to account for the observed Gaussian decay reflecting an inhomogeneous broadening of the $|g\rangle \to |s\rangle$ transition, the exponential decay term $e^{-\gamma_0 t}$ in Eq. (5) is replaced with a Gaussian decay $e^{-\frac{1}{2}(t/\gamma_0)^2}$, with $\gamma_0 = 53\,\mu$s. The simulation is performed from Eq. (7) and uses the following measured values: The write pulse has a Rabi frequency of $\Omega_W = 25.1$ MHz and a FWHM of 15 ns, detuned by -40 MHz from the $|e\rangle \to |g\rangle$ transition. The peak Rabi frequency of the read pulse is at 23.5 MHz with a FWHM of 35 ns. We take $d_w = 7.5$ and $d_r = 5$. An error of 10% on the Rabi frequencies, pulse widths, optical depths and spin coherence time was assumed in order to obtain the bounds on the simulation.

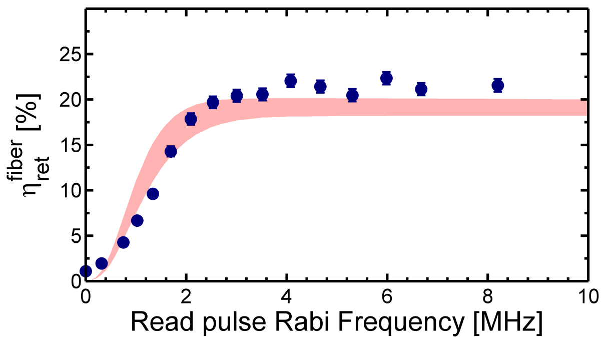

Supplementary Figure 2: **Fiber-coupled conditional retrieval efficiency $\eta_{\rm ret}^{\rm fiber}$ vs read Rabi frequency Ω_R.** Experimental data (blue dots) are compared with numerical simulations (red shaded area). The simulation is performed from Eq. (7) and uses the values of the write pulse presented in the caption of Supplementary Figure 1. The peak Rabi frequency of the read pulse is varied and its FWHM is $1.27\,\mu$s. It has a delay of $2.16\,\mu$s from the write peak frequency. We take $d_w = 4.4$ and $d_r = 2.9$. An error of 10% on the Rabi frequencies, pulse widths, optical depths and spin coherence time was assumed in order to obtain the bounds on the simulation.

Supplementary Notes

Supplementary Note 1: CHARACTERIZATION OF THE QUANTUM MEMORY

In this section, we present additional experimental characterizations of the quantum memory and compare them to simulations based on the formalism introduced in the former section. Note first that for simulating the experimental data, we use the following conventions for the write (read) Rabi frequencies and optical depths: $\Omega_W = 2\bar{\Omega}_W = \langle e|\mathbf{d}\cdot\mathbf{E}_W|g\rangle$ ($\Omega_R = 2\bar{\Omega}_R$) with $\mathbf{d}$ the dipole operator and $\mathbf{E}_W$ the electric field amplitude of the write pulse and $d_w = 2\bar{d}_w$ ($d_r = 2\bar{d}_r$) such that the attenuation of the outgoing light intensity decreases as $I(L) = e^{-d_w(d_r)}I(0)$.

Supplementary Figure 1 shows the fiber-coupled conditional retrieval efficiency as a function of the delay between the write and read pulses. To account for the observed Gaussian decay of the retrieval efficiency, reflecting an inhomogeneous broadening of the $|g\rangle \rightarrow |s\rangle$ transition, all the simulations are performed by replacing the exponential decay term $e^{-\gamma_0 t}$ in Eq. (5) with a Gaussian decay $e^{-\frac{1}{2}(t/\gamma_0)^2}$.

To optimize the retrieval efficiency, we scanned the intensity of the driving read pulse for fixed photon durations. For short photon durations, we observe the expected Rabi oscillations in the retrieval efficiency vs. read pulse power [2]. If we generate photons with durations longer than the natural decay time, the oscillations are damped and the efficiency approaches a constant value for high read pulse intensities. Supplementary Figure 2 shows such an example of the fiber-coupled conditional retrieval efficiency as a function of the Rabi frequency Ω_R of the driving read pulse. Note that the results of Supplementary Figure 1 and Supplementary Figure 2 have been taken with slightly modified setups, hence the differing optical depths.

Supplementary Note 2: PRINCIPLE OF NUMERICAL SIMULATION

Here, we explain how one can compute the read photon properties conditioned on a write emission. We begin by solving the dynamics of the write field and the spin-wave. We then provide the explicit expression of the read field before showing how it can be used to obtain the conditional read photon characteristics.

A. Write field and spin-wave expressions

Working with a Λ-scheme for a three level system, we consider a writing pulse $\bar{\Omega}_W(t)$ detuned by Δ from the $|g\rangle \rightarrow |e\rangle$ transition. The $|e\rangle \rightarrow |s\rangle$ transition is characterized by an optical depth $\bar{d}_w$. γ_{es} describes the decay of coherence in the $|e\rangle \rightarrow |s\rangle$ transition, and γ_0 describes the decay of the $|g\rangle \rightarrow |s\rangle$ coherence. In the limit where the write field $\hat{\mathcal{E}}_w$ is slowly varying, propagating in a pencil-shaped atomic ensemble in which the $|g\rangle \rightarrow |e\rangle$ transition is driven by a off-resonant write pulse of duration τ_W satisfying $\gamma_{es}\tau_W\bar{d}_w \ll 1$, and also operating in the regime where $\Delta \gg |\bar{\Omega}_W|, \gamma_{es}$, the Raman scattering process results in the emission of a write field and the creation of a correlated spin-wave $\hat{S}$, whose dynamics are described with the Heisenberg-Langevin equations

$$\begin{aligned} c\partial_{z'}\hat{\mathcal{E}}_w &= i\chi\hat{S}^\dagger \\ \partial_{t'}\hat{S}^\dagger &= -\Gamma_S\hat{S}^\dagger - i\chi\hat{\mathcal{E}}_w + \hat{F}_S^\dagger \end{aligned} \tag{1}$$

Here, we introduced shifted coordinates $z' = z$ and $t' = t - z/c$. $\chi(t) = (\sqrt{\bar{d}_w\gamma_{es}c/L})\frac{\bar{\Omega}_W(t)}{\Delta}$, where L is the length of the atomic medium, $\Gamma_S(t) = \gamma_S(t) + i\delta_S(t)$, $\gamma_S(t) = \gamma_0 + \gamma_{es}\frac{|\bar{\Omega}_W(t)|^2}{\Delta^2}$, $\delta_S(t) = -\frac{|\bar{\Omega}_W(t)|^2}{\Delta}$ and $\hat{F}_S$ is the Langevin noise operator for the write process. The commutation relations for the relevant operators are given by

$$\begin{aligned} &[\hat{\mathcal{E}}_w(z,t), \hat{\mathcal{E}}_w^\dagger(z',t')] = L\delta[z - z' - c(t-t')] \\ &[\hat{S}(z,t), \hat{S}^\dagger(z',t)] = L\delta(z - z') \\ &\langle \hat{F}_S(z,t)\hat{F}_S^\dagger(z',t')\rangle = 2\gamma_S L\delta(z-z')\delta(t-t') \\ &\langle \hat{F}_S^\dagger(z,t)\hat{F}_S(z',t')\rangle = 0 \end{aligned} \tag{2}$$

The equations of motion can be solved as shown in Ref. [1]. The solutions for the spin-wave and write field are

$$\begin{aligned} \hat{S}^\dagger(z',t') &= e^{-\Gamma(t')}\hat{S}^\dagger(z',0) \\ &+ \int_0^{t'} e^{-[\Gamma(t')-\Gamma(t'')]}\hat{F}_S^\dagger(z',t'')dt'' \\ &- i\int_0^{t'} \chi(t'')e^{-[\Gamma(t')-\Gamma(t'')]}H(z',0,t',t'')\hat{\mathcal{E}}_w(0,t'')dt'' \\ &+ e^{-\Gamma(t')}\int_0^{z'} G_s(z',z'',t',0)\hat{S}^\dagger(z'',0)dz'' \\ &+ \int_0^{t'} e^{-[\Gamma(t')-\Gamma(t'')]}\int_0^{z'} G_s(z',z'',t',t'')\hat{F}_S^\dagger(z'',t'')dz''dt'' \end{aligned} \tag{3}$$

and

$$\begin{aligned}
\hat{\mathcal{E}}_w(z',t') &= \hat{\mathcal{E}}_w(0,t') \\
&+ i(\chi(t')/c)e^{-\Gamma(t')}\int_0^{z'} H(z',z'',t',0)\hat{S}^\dagger(z'',0)dz'' \\
&+ i(\chi(t')/c)\int_0^{t'} e^{-[\Gamma(t')-\Gamma(t'')]}\int_0^{z'} H(z',z'',t',t'')\hat{F}_S^\dagger(z'',t'')dz''dt'' \\
&+ (\chi(t')/c)\int_0^{t'} \chi(t'')e^{-[\Gamma(t')-\Gamma(t'')]}G_e(z',0,t',t'')\hat{\mathcal{E}}_w(0,t'')dt'', \qquad (4)
\end{aligned}$$

where

$$\begin{aligned}
H(z',z'',t',t'') &= I_0\left(2\sqrt{[g(t')-g(t'')]\frac{z'-z''}{c}}\right) \\
G_s(z',z'',t',t'') &= \sqrt{\frac{g(t')-g(t'')}{c(z'-z'')}}I_1\left(2\sqrt{[g(t')-g(t'')]\frac{z'-z''}{c}}\right) \\
G_e(z',z'',t',t'') &= \left(\frac{c(z'-z'')}{g(t')-g(t'')}\right)G_s(z',z'',t',t'').
\end{aligned}$$

Here, $I_n(x)$ refers to the modified Bessel function of the first kind, and here we have defined $\Gamma(t) = \int_0^t \Gamma_S(t)dt$, $g(t) = \int_0^t \chi(t')^2 dt'$.

B. Read field expression

During the retrieval process, a read pulse with Rabi frequency $\bar{\Omega}_R(t)$ is applied resonant with the $|e\rangle \to |s\rangle$ transition, converting the spin-wave in the atomic medium into a read field resonant with the $|e\rangle \to |g\rangle$ transition. The $|e\rangle \to |g\rangle$ transition is characterized by an optical depth $\bar{d}_r$. γ_{eg} describes the decay of coherence in the $|e\rangle \to |g\rangle$ transition. Following similar arguments as Ref. [1], we can find the explicit expression of the read field $\hat{\mathcal{E}}_r$ as a function of the spin-wave resulting from the write process. Here, we consider a write emission at time t_i, and a non-zero read field at time t_d after the write pulse ends. For the retrieval, the read field is emitted backwards, towards the z=0 position of the atomic medium. In the regime of $\bar{d}_r \gg 1$ and sufficiently long read field duration $\tau_r \gg \frac{1}{\gamma_{eg}\bar{d}_r}$,

$$\begin{aligned}
&\hat{\mathcal{E}}_r(0, t = t_d + \xi) \\
&= -\frac{\bar{\Omega}_R(t)}{g\sqrt{N}}e^{-\gamma_0(t-\xi)}\int_{c\Delta\tau(t,\xi)}^{L+c\Delta\tau(t,\xi)} \frac{1}{\sqrt{2\pi}\Delta l(t,\xi)}\exp\left[-\frac{1}{2}\left(\frac{L-z}{\Delta l(t,\xi)}\right)^2\right]\hat{S}\left(L-z+c\Delta\tau(t,\xi),\xi\right)dz \\
&- \frac{\bar{\Omega}_R(t)}{g\sqrt{N}}\int_\xi^t\int_{c\Delta\tau(t,\xi))}^{L+c\Delta\tau(t,\xi)} \frac{e^{-\gamma_0(t-t')}}{\sqrt{2\pi}\Delta l(t,t')}\exp\left[-\frac{1}{2}\left(\frac{L-z}{\Delta l(t,t')}\right)^2\right]\times \\
&\left[\hat{F}_S(L-z+c\Delta\tau(t,t'),t')\right. \\
&\left.+ i\frac{\Delta l^2(t,t') + (L-z)(2c(\Delta\tau(t,t')) + L - z)}{4\gamma_{eg}c^2(\Delta\tau(t,t'))^2}\bar{\Omega}_R(t')\hat{F}_P(L-z+c\Delta\tau(t,t'),t')\right]dz \\
&+ \frac{i}{g\sqrt{N}}e^{-\gamma_0 t}\hat{F}_P(0,t), \qquad (5)
\end{aligned}$$

where the commutation relations are

$$\begin{aligned}
&[\hat{\mathcal{E}}_r(z,t), \hat{\mathcal{E}}_r^\dagger(z',t')] = L\delta[z - z' - c(t-t')] \\
&\langle\hat{F}_P(z,t)\hat{F}_P^\dagger(z',t')\rangle = 2\gamma_{eg}L\delta(z-z')\delta(t-t') \\
&\langle\hat{F}_P^\dagger(z,t)\hat{F}_P(z',t')\rangle = 0.
\end{aligned}$$

Here, $\Delta\tau(t,t') = \frac{L}{d_r \gamma_{eg} c}\int_{t'}^{t} \bar{\Omega}_R^2(t'')dt''$, and $\Delta l(t,t') = \sqrt{\frac{2Lc}{d_r}\Delta\tau(t,t')}$. g refers to the coupling constant between a single atom and a single read photon, and N corresponds to the number of interacting atoms. This can be expressed as $g^2 N = \frac{d_r \gamma_{eg} c}{L}$. ξ indicates a suitable time after the write pulse has ended, and where the read pulse is considered to begin, so as to perform the numerical integration for the retrieval. $\hat{F}_P$ is the Langevin noise operator for the retrieve process.

C. Conditional retrieval efficiency

Equipped with the above expressions for the optical fields and spin-wave, we can compute the expectation of read photon emissions conditioned on the emission of a write photon from

$$\eta_{r|w} = \frac{c}{L}\frac{\int\int \langle \hat{\mathcal{E}}_w^\dagger(L,t_i)\hat{\mathcal{E}}_r^\dagger(0,t)\hat{\mathcal{E}}_r(0,t)\hat{\mathcal{E}}_w(L,t_i)\rangle dt_i dt}{\int \langle \hat{\mathcal{E}}_w^\dagger(L,t_i)\hat{\mathcal{E}}_w(L,t_i)\rangle dt_i}. \tag{6}$$

Evaluating the expression $\langle \hat{\mathcal{E}}_w^\dagger(L,t_i)\hat{\mathcal{E}}_r^\dagger(0,t)\hat{\mathcal{E}}_r(0,t)\hat{\mathcal{E}}_w(L,t_i)\rangle$ requires the expression in Eq. (4) and only the first term in Eq. (5), which one in turn develops using Eq. (3). A tedious but straightforward computation then results in 12 nonzero terms, of which 3 terms are 4-point noise correlators. Such 4-point noise correlations can be evaluated with use of Isserlis' theorem, which allows a decomposition into 2-point noise correlators for Gaussian random variables.

This gives, for example,

$$\begin{aligned}
\langle \hat{F}_S(z_1,t_1)\hat{F}_S^\dagger(z_2,t_2)\hat{F}_S(z_3,t_3)\hat{F}_S^\dagger(z_4,t_4)\rangle &= \langle \hat{F}_S(z_1,t_1)\hat{F}_S^\dagger(z_2,t_2)\rangle\, \langle \hat{F}_S(z_3,t_3)\hat{F}_S^\dagger(z_4,t_4)\rangle \\
&+ \langle \hat{F}_S(z_1,t_1)\hat{F}_S(z_3,t_3)\rangle\, \langle \hat{F}_S^\dagger(z_2,t_2)\hat{F}_S^\dagger(z_4,t_4)\rangle \\
&+ \langle \hat{F}_S(z_1,t_1)\hat{F}_S^\dagger(z_4,t_4)\rangle\, \langle \hat{F}_S^\dagger(z_2,t_2)\hat{F}_S(z_3,t_3)\rangle,
\end{aligned}$$

where only the first term survives since the normal-ordered 2-point noise correlators are zero.

Finally, from the coupling efficiency of the read emission into the first fiber η_{fiber}, we can reproduce the fiber-coupled conditional retrieval efficiency $\eta_{\text{ret}}^{\text{fiber}}$ using

$$\eta_{\text{ret}}^{\text{fiber}} = \eta_{r|w}\eta_{\text{fiber}}, \tag{7}$$

valid in the low photon number regime ($\eta_{r|w} \ll 1$).

D. Read photon shape

The explicit expression of the fields also allows us to predict the temporal dependence of the conditional read emission, in a similar way as above. In particular, the conditional read photon flux inside the first fiber is given by

$$n_r^{cond}(t) = \frac{c}{L}\frac{\int \langle \hat{\mathcal{E}}_w^\dagger(L,t_i)\hat{\mathcal{E}}_r^\dagger(0,t)\hat{\mathcal{E}}_r(0,t)\hat{\mathcal{E}}_w(L,t_i)\rangle dt_i}{\int \langle \mathcal{E}_w^\dagger(L,t_i)\mathcal{E}_w(L,t_i)\rangle dt_i}\eta_{\text{fiber}}. \tag{8}$$

Supplementary Note 3: QUANTUM FEATURES AND PURITY OF THE READ PHOTONS

To prove that the conditional read emission takes the form of single photons, we have measured the second order autocorrelation function conditioned on the detection of a write photon. Assuming that the write-read photon pairs are described by a two-mode squeezed vacuum state, an explicit expression of the conditional second order autocorrelation function can be derived in a non-perturbative way while taking the detector imperfections into account (non-unit, noisy and non-photon number resolving detectors), see Eqs. (24)-(25) in Ref. [3]. The agreement between this model and the experimental data shows that the heralded second order autocorrelation function is mainly limited by dark counts, see Fig. 4(a) in the main text.

To conclude about the purity of the heralded read emission, we have also measured the (unconditional) second order autocorrelation function. Assuming again that the state of the write-read photon pairs corresponds to a two-mode squeezed vacuum, the exact expression of the second order autocorrelation function can be derived taking the detector imperfection into account, see formula $\tilde{g}^{(2)}_{\sum_n a_n}$ after Eq. (39) in Ref. [3]. In particular, in the absence of noise and for small detection efficiencies, the auto-correlation function is given by $1+1/K$, i.e. depends on the number of modes K. The full (blue) and dashed (purple) lines in Fig. 4 (b) of the main text are obtained by assuming that the read photons are emitted in a single mode and in two possible modes respectively (with the detector imperfections). This shows that the read emission is single mode and together with the result of the conditional auto-correlation measurement, we conclude that the heralded emission is close to a pure single photon.

Supplementary Note 4: COMPARISON WITH OTHER APPROACHES

In this section, we briefly compare our approach with other investigated experimental platforms to generate ultra-narrowband photons.

Cavity-enhanced spontaneous parametric down conversion (SPDC) has been proven to allow for generation of single photons pairs at high rates and spectral brightness [4–6]. However, single photons with ultra-narrow bandwidth in the range of 100 kHz have not been demonstrated yet via SPDC. The narrowest single photons in single mode generated by SPDC so far has a bandwidth of around 2 MHz [6]. Although narrower linewidths are in principle possible, it will be challenging to reach a linewidth as narrow as 100 kHz, because of the limited cavity finesse achievable due to the optical loss in the non-linear crystal. Also the tunability of the photon waveshape, as demonstrated in our manuscript, would be very challenging using SPDC, as it would require a dynamic and highly accurate control of the cavity finesse. To the best of our knowledge, such tunable SPDC experiments have not been presented yet. Finally, the SPDC approach alone doesn't enable a controllable emission time for the heralded photon.

Another common approach is based on spontaneous four wave mixing (SFWM) by which bi-photon coherence times up to 1 μs could be obtained [7]. Although that approach offers a tunability of the photon duration of about one order of magnitude [8], i.e. two orders of magnitude less than demonstrated here, photon durations are intrinsically limited by the coherence times of the involved levels, which might be limited by dephasing due to a magnetic gradient in the experiment. Also the delay between the generated anti-Stokes and the Stokes photons relies on slow light via electromagnetically induced transparency (EIT). In our case, the magnetic field is switched off during the experiment, leading to much longer coherence time. Also, the achievable delay between the two photons depends only on the ground state coherence time. Finally, in contrast to the FWM case we also don't need to deal with optical precursors.

Single photons of tunable length have also been produced by spontaneous Raman transitions in trapped single ions [9]. Here, the photon length could be varied depending on the laser-controlled Raman transition rate. The longest coherence time observed was 1.6 μs limited by the natural lifetime of the particular transition in $^{40}Ca^{+}$.

Finally, there have been also attempts to realize DLCZ-type quantum memories in hot atomic vapors. But their performance is limited due to high background noise and collisional decoherence during the write and read-out process [10]. A possible route to overcome these problems could be the use of micro-cells [11]. However, the generation of ultra-narrow single photons still has to be demonstrated using that approach.

In other systems like in rare-earth doped solids, DLCZ-like experiments are currently being investigated [12–16]. Whether ultra-narrow single photons with widely tunable waveshape can be produced with that approach is still an open question and needs to be further investigated.

Supplementary References

[1] André, A. *Nonclassical states of light and atomic ensembles: Generation and New Applications.* PhD thesis, Harvard University (2005).

[2] Mendes, M. S., Saldanha, P. L., Tabosa, J. W. R. & Felinto, D. Dynamics of the reading process of a quantum memory. *New Journal of Physics* **15**, 075030 (2013).

[3] Sekatski, P. *et al.* Detector imperfections in photon-pair source characterization. *Journal of Physics B: Atomic, Molecular and Optical Physics* **45**, 124016 (2012).

[4] Bao, X.-H. *et al.* Generation of Narrow-Band Polarization-Entangled Photon Pairs for Atomic Quantum Memories. *Physical Review Letters* **101**, 190501 (2008).

[5] Haase, A., Piro, N., Eschner, J. & Mitchell, M. W. Tunable narrowband entangled photon pair source for resonant single-photon single-atom interaction. *Optics Letters* **34**, 55–57 (2009).

[6] Fekete, J., Rieländer, D., Cristiani, M. & de Riedmatten, H. Ultranarrow-Band Photon-Pair Source Compatible with Solid State Quantum Memories and Telecommunication Networks. *Physical Review Letters* **110**, 220502 (2013).

[7] Zhao, L. *et al.* Photon pairs with coherence time exceeding 1 μs. *Optica* **1**, 84–88 (2014).

[8] Du, S., Kolchin, P., Belthangady, C., Yin, G. Y. & Harris, S. E. Subnatural Linewidth Biphotons with Controllable Temporal Length. *Physical Review Letters* **100**, 183603 (2008).

[9] Almendros, M. *et al.* Bandwidth-Tunable Single-Photon Source in an Ion-Trap Quantum Network. *Physical Review Letters* **103**, 213601 (2009).

[10] Manz, S., Fernholz, T., Schmiedmayer, J. & Pan, J.-W. Collisional decoherence during writing and reading quantum states. *Physical Review A* **75**, 040101 (2007).

[11] Borregaard, J. *et al.* Scalable photonic network architecture based on motional averaging in room temperature gas. *Nature Communications* **7**, 11356 (2016).

[12] Ledingham, P. M., Naylor, W. R. & Longdell, J. J. Nonclassical photon streams using rephased amplified spontaneous emission. *Physical Review A* **81**, 012301 (2010).

[13] Sekatski, P., Sangouard, N., Gisin, N., de Riedmatten, H. & Afzelius, M. Photon-pair source with controllable delay based on shaped inhomogeneous broadening of rare-earth-metal-doped solids. *Physical Review A* **83**, 053840 (2011).

[14] Ledingham, P. M., Naylor, W. R. & Longdell, J. J. Experimental Realization of Light with Time-Separated Correlations by Rephasing Amplified Spontaneous Emission. *Physical Review Letters* **109**, 093602 (2012).

[15] Beavan, S. E., Hedges, M. P. & Sellars, M. J. Demonstration of Photon-Echo Rephasing of Spontaneous Emission. *Physical Review Letters* **109**, 093603 (2012).

[16] Ferguson, K. R., Beavan, S. E., Longdell, J. J. & Sellars, M. J. Generation of Light with Multimode Time-Delayed Entanglement Using Storage in a Solid-State Spin-Wave Quantum Memory. *Physical Review Letters* **117**, 020501 (2016).
